# From shadows to sunlight: Rethinking Intraprofessional collaboration beyond the cave

**DOI:** 10.1111/medu.15659

**Published:** 2025-03-10

**Authors:** Rosalin van Schie, Ester Coolen, Bart Thoonen

**Affiliations:** ^1^ Department of Primary and Community Care Radboud University Medical Centre Nijmegen The Netherlands; ^2^ Department of Paediatrics Radboud University Medical Centre Amalia Children's Hospital Nijmegen The Netherlands

## Abstract

Schie et al. argue that medical education must move beyond entrenched hierarchies and siloed thinking. Like Plato's cave, escaping illusions enables collaboration, recognizing all roles to improve patient care and learning.

In this issue of *Medical Education*, Wong et al. illustrate how taking the system for granted can blind us to its negative and self‐reinforcing impact on intraprofessional education.^1^ The authors argue that we must recognise our ‘blindness’ to the contextual forces at play, as failing to recognise them may inadvertently reinforce boundaries in intraprofessional collaboration. Their key messages reflect Plato's Allegory of the Cave,^2^ suggesting that adopting a critical perspective can free us from the cave, beyond managing power dynamics, enabling us to challenge reality and develop a deeper understanding of the socio‐medical landscape. This is a challenge that we aim to explore further in this commentary.

*Plato allegory in brief:*

*In Plato's allegory, the prisoners in the cave represent individuals who are limited to a restricted understanding of the world, seeing only projected shadows on the wall. One prisoner is freed and exposed to the outside world, representing the individual who gains true knowledge. Initially, the freed prisoner struggles with the overwhelming truth, but through gradual adaptation, he comes to understand the true reality beyond the cave. Upon returning to the dark cave, he is blinded by the darkness after having seen the light, making it difficult for him to convince the remaining prisoners that the world outside the cave is real. The other prisoners do not believe his insights and even ridicule him. They cannot comprehend that their reality of shadows is merely an illusion, and they might even react with hostility if he tries to free them*.

In the early stages of medical education, students often begin their training in a metaphorical ‘cave’. They are exposed to a simplified, structured understanding of medicine, primarily focusing on medical textbooks, clinical guidelines and science. These early experiences can be seen as ‘shadows’ of the broader, more complex realities of medical practice. Just as the prisoners believe the shadows are the entirety of reality, a medical student or even young professional might believe that mastering these basic skills and knowledge during their medical education is all that is required for successful practice. The *shadows* may create a false sense of security—or the illusion of knowledge—that does not align with the complexities of real‐world patient‐centered care.

“The *shadows* may create a false sense of security—or the illusion of knowledge.”

As physicians advance in their medical careers, the illusion that medical‐technical knowledge and skills alone guarantee optimal patient care appears to be maintained deeply ingrained and dominant. Wong et al. clearly illustrate that specialist physicians (SPs), relying primarily on a knowledge‐driven hierarchy, guide generalist family physicians (FPs), shaping and sustaining professional interactions. There is no true collaborative partnership in which FPs' expertise—rooted in a holistic approach and extensive knowledge of the patient and their context—is fully utilised. On the contrary, as Wong et al. describe, under high workload and patient volume, decisions are predominantly made based on SPs' medical‐technical knowledge rather than through joint efforts to find efficient solutions for increasing patient pressure. As such, power dynamics, driven by a knowledge based hierarchy, sustain a system in which groups adhere to entrenched socialised routines, making it challenging for either group to escape the proverbial cave.

“Groups adhere to entrenched socialised routines, making it challenging for either group to escape the proverbial cave.”

So why is it that staying in the cave is no longer a viable option? There are multiple pressing reasons to escape. The most critical reasons include the recognition that most adverse events in patient care result from failures in collaboration rather than from individual medical‐technical competence, alongside increasing health care demands, rising costs and growing workloads.^3^, ^4^ Addressing these complex challenges requires integrated expertise, where contributions from multiple domains are indispensable.

Given the urgency to escape, what must be done to begin the journey out of the cave—and, more importantly, to stay outside? Given the deeply entrenched social routines, we propose measures from the perspectives of the profession, medical education programmes and professional development of (future) health care professionals.

“We propose measures from the perspectives of the profession, medical education programmes and professional development of (future) health care professionals.”

From the profession's perspective, societal recognition of each profession's expertise and their equal roles are essential. Professional medical associations are key in acknowledging the current power dynamics and in facilitating a transition from collaboration from within separate communities of practice, towards collaborative care across a landscape of practice.^5^

From the perspective of medical education, reassessing the current mission and vision of training programmes is required. One way to broaden our minds beyond the cave is by using Biesta et al.’s framework, which balances three interdependent goals of (medical) education: competency development (qualification), professional community integration (socialisation) and the cultivation of autonomy and professional identity (subjectification).^6^ Incorporating these goals into training programmes signals that these are conditional in shaping medical curricula.

For the professional development of (future) health professionals, inspirational guides on the journey are essential. As Plato's allegory reminds us, the transition can be disorienting at first and there is a risk of getting blinded by the darkness after having seen the light. Supervisors' positive reinforcement and open appreciation of other professionals can support learners adapt to new ways of collaborating. Therefore, positive role models are key to intraprofessional education, while negative modelling can severely hinder collaborative learning.^7^

An additional and powerful catalyst for intraprofessional learning occurs when learners personally experience the benefits and joy of stepping into the sunlight. Collaborative cross‐setting learning can initiate astoundment and awareness about each other's roles and contexts creating new opportunities to streamline collaboration in patient care, benefiting both patients and professionals.^8^ These experiences can shape (future) professionals into ambassadors and role models, fostering a collaborative mindset in the health care workforce.

“Collaborative cross‐setting learning can initiate astoundment and awareness about each other's roles and contexts creating new opportunities.”

Thus, standing in the sunlight together represents the realisation of medicine as a vocation—where physicians see themselves not only as medical‐technical experts, but as healers, advocates, and lifelong learners collaborating in patient care. In the sunlight, no single dominant hue prevails; rather, the full spectrum forms a complementary whole, reflecting the diverse expertise required in health care. If we collectively embrace this vision, we will be able to travel in and out of the cave in order to inspire others to embark on their own journey.

“In the sunlight, no single dominant hue prevails; rather, the full spectrum forms a complementary whole.”

## AUTHOR CONTRIBUTIONS

Rosalin van Schie led the conceptualisation of the commentary, in close collaboration with Ester Coolen and Bart Thoonen. Rosalin van Schie was primarily responsible for drafting and developing the manuscript, structuring the arguments and integrating revisions. Ester Coolen and Bart Thoonen provided critical feedback, contributed to revisions and helped refine the final version. All authors reviewed and approved the final manuscript and meet the ICMJE criteria for authorship.

## CONFLICT OF INTEREST STATEMENT

The authors declare no competing interests.

## References

[1] Wong R , Whitehead CR . Exploring how structural forms of power shape the training of intraprofessional collaboration between family physicians and specialty physicians in outpatient workplace settings. Med Educ. 2025;59. 10.1111/medu.15607 39815955

[2] Plato . The allegory of the cave [Sheehan T, translator]. *Republic*, VII, 514 a, 2 to 517 a. Vol. 7; 2017 [cited 2025 Feb 10]. Available from: https://web.stanford.edu/class/ihum40/cave.pdf

[3] Sutcliffe KM , Lewton E , Rosenthal MM . Communication failures: an insidious contributor to medical mishaps. Acad Med. 2004;79(2):186‐194. doi:10.1097/00001888-200402000-00019 14744724

[4] Looman N , Fluit C , van Wijngaarden M , de Groot E , Dielissen P , van Asselt D , de Graaf J , Scherpbier‐ Haan N. Chances for learning intraprofessional collaboration between residents in hospitals Med Educ 2020;54(12):1109‐1119. doi. 10.1111/medu.14279 32564390 PMC7754101

[5] Stalmeijer RE , Varpio L . The wolf you feed: challenging intraprofessional workplace‐based education norms. Med Educ. 2021;55(8):894‐902. doi:10.1111/medu.14520 33651450 PMC8359828

[6] Biesta G . Risking ourselves in education: qualification, socialization, and subjectification revisited. Educ Theory. 2020;70(1):89‐104. doi:10.1111/edth.12411

[7] Teheux L , Kuijer‐Siebelink W , Bus LL , Draaisma JMT , Coolen EHAJ , van der Velden JAEM . Unravelling underlying processes in intraprofessional workplace learning in residency. Med Educ. 2024;58(8):939‐951. doi:10.1111/medu.15271 37990961

[8] van Schie R , Coolen E , van Tuijl A , van der Velden J , Thoonen B , Scherpbier‐de Haan N . Intraprofessional boundary crossing in acute paediatric care: a rich journey for general practice and paediatric residents. Med Educ. 2024;58(11):1350‐1360. doi:10.1111/medu.15423 38741165

